# Long‐term, but not short‐term high‐fat diet induces fiber composition changes and impaired contractile force in mouse fast‐twitch skeletal muscle

**DOI:** 10.14814/phy2.13250

**Published:** 2017-04-13

**Authors:** Hiroaki Eshima, Yoshifumi Tamura, Saori Kakehi, Nagomi Kurebayashi, Takashi Murayama, Kyoko Nakamura, Ryo Kakigi, Takao Okada, Takashi Sakurai, Ryuzo Kawamori, Hirotaka Watada

**Affiliations:** ^1^ Department of Metabolism & EndocrinologyJuntendo University Graduate School of MedicineTokyoJapan; ^2^ Sportology CenterJuntendo University Graduate School of MedicineTokyoJapan; ^3^ Department of Cellular and Molecular PharmacologyJuntendo University Graduate School of MedicineTokyoJapan; ^4^ Department of PhysiologyJuntendo University Graduate School of MedicineTokyoJapan; ^5^ Center for Therapeutic Innovations in DiabetesJuntendo University Graduate School of MedicineTokyoJapan; ^6^ Center for Molecular DiabetologyJuntendo University Graduate School of MedicineTokyoJapan

**Keywords:** High‐fat diet, insulin resistance, muscle contractile force, muscle fiber composition

## Abstract

In this study, we investigated the effects of a short‐term and long‐term high‐fat diet (HFD) on morphological and functional features of fast‐twitch skeletal muscle. Male C57BL/6J mice were fed a HFD (60% fat) for 4 weeks (4‐week HFD) or 12 weeks (12‐week HFD). Subsequently, the fast‐twitch extensor digitorum longus muscle was isolated, and the composition of muscle fiber type, expression levels of proteins involved in muscle contraction, and force production on electrical stimulation were analyzed. The 12‐week HFD, but not the 4‐week HFD, resulted in a decreased muscle tetanic force on 100 Hz stimulation compared with control (5.1 ± 1.4 N/g in the 12‐week HFD vs. 7.5 ± 1.7 N/g in the control group; *P *<* *0.05), whereas muscle weight and cross‐sectional area were not altered after both HFD protocols. Morphological analysis indicated that the percentage of type IIx myosin heavy chain fibers, mitochondrial oxidative enzyme activity, and intramyocellular lipid levels increased in the 12‐week HFD group, but not in the 4‐week HFD group, compared with controls (*P *<* *0.05). No changes in the expression levels of calcium handling‐related proteins and myofibrillar proteins (myosin heavy chain and actin) were detected in the HFD models, whereas fast‐troponin T‐protein expression was decreased in the 12‐week HFD group, but not in the 4‐week HFD group (*P *<* *0.05). These findings indicate that a long‐term HFD, but not a short‐term HFD, impairs contractile force in fast‐twitch muscle fibers. Given that skeletal muscle strength largely depends on muscle fiber type, the impaired muscle contractile force by a HFD might result from morphological changes of fiber type composition.

## Introduction

Metabolic disorders, such as insulin resistance and type 2 diabetes mellitus, can cause a decrease in muscle strength and loss of muscle mass (Park et al. 2006, 2009; Zhou et al. 2007; Lee et al. 2011). In addition, previous studies have demonstrated that decreased muscle strength is observed in obese young adults (Maffiuletti et al. 2007) and older adults (Zoico et al. 2004; Tomlinson et al. 2014a). The decreased muscle strength observed in obese subjects may be caused by decreased muscle quantity and/or quality, which is calculated as muscle strength per unit of muscle mass (Tomlinson et al. 2014b). A recent study demonstrated that muscle strength normalized to fiber size was substantially lower in obese older adults (Choi et al. 2016). This result suggests that decreased muscle strength in obese subjects is caused by decreased muscle contractile force. However, the underlying mechanism of the association between obesity and a decrease in muscle contractile force has not been fully elucidated.

Although maximal contractile force largely depends on fast‐twitch fibers rather than slow‐twitch fibers (Bottinelli et al. 1991, 1994), a high‐fat diet (HFD)‐induced rodent obesity model did not demonstrate a decreased muscle contractile force in fast‐twitch dominant muscles. For example, mice fed a HFD for 3 weeks (Thomas et al. 2014) or 8 weeks (Shortreed et al. 2009) did not have significant impairment of the contractile force of fast‐twitch dominant muscles. Another study also showed that 5 weeks on a HFD reduced tetanic contractile force only in slow‐twitch dominant muscles, but not in fast‐twitch dominant muscles (Ciapaite et al. 2015). Therefore, being on a HFD for only several weeks do not significantly alter muscle contractile force in fast‐twitch dominant muscles. However, the effects of being fed a HFD for longer than 8 weeks on the muscle contractile force of fast‐twitch dominant muscles has not yet been elucidated.

Skeletal muscle contraction is regulated by several functional cellular compartments, such as action potential, Ca^2+^ release, cross‐bridge cycling, energy supply, and muscle fiber type composition (Dux 1993; Allen et al. 2008; Schiaffino and Reggiani 2011; Eshima et al. 2014). In particular, contractile force largely depends on fiber type and metabolic profile of mammalian skeletal muscle (Barany 1967). In addition, myosin isoform is a major factor thought to contribute to differences in muscle contractile force in different fiber types (Pette and Staron 2000; Baylor and Hollingworth 2003). For example, mammalian skeletal muscle fibers express either slow‐type myosin, or three fast‐type myosin isoforms: types IIa, IIx, and IIb (Gilliver et al. 2009). The contractile forces produced by the different MHC‐type fibers are in the following order: slow < IIa < IIx < IIb (Bottinelli et al. 1991, 1996; Schiaffino and Reggiani 2011). Although a recent study showed that 16 weeks on a HFD decreased the number of type IIb fibers and increased the number of type IIa fibers without assessing muscle contraction force (Mastrocola et al. 2015), the effect of a long‐term HFD on muscle fiber composition and muscle contractile force have not yet been fully elucidated.

Based on this information, we hypothesized that altered muscle fiber composition and impaired contractile force in fast‐twitch dominant muscle after a long‐term HFD, but not short‐term HFD mice. Thus, we investigated the effect of a short‐term and long‐term HFD on the contractile force and morphological changes of muscles, including muscle fiber type composition in fast‐twitch skeletal muscle.

## Materials and Methods

### Animals

Male C57BL6J mice were housed in cages in a temperature‐controlled room under a 12 h light–dark cycle. During a 1‐week adaptation period, all mice were fed a standard chow and water ad libitum. After an initial acclimatization period, animals (8 weeks of age) were randomly assigned to either the group fed a HFD (D12492, Research Diets, New Brunswick, NJ) containing 20% protein, 60% fat, 20% carbohydrate, or the grouped fed standard chow. After a short‐term (4‐week) or long‐term (12‐week) HFD, we performed blood and muscle sampling for further analysis, as described below. All animal experiments in this study were approved by the Animal Experimental Committee of Juntendo University.

### Assessment of glucose metabolism

The intraperitoneal glucose tolerance test (IPGTT) was performed on mice fasted overnight (16 hrs) after 3 weeks or 11 weeks of diet intervention. Glucose was injected intraperitoneally (0.5 g/kg body weight) and blood glucose was assessed by tail bleeds at 0, 15, 30, 60, 90, 120, and 150 min. Fasted plasma insulin were measured using an ELISA kit (Morinaga Co., Kanagawa, Japan) in according to the manufacturer's protocol. The homeostasis model assessment of insulin resistance (HOMA‐IR) was calculated as previously described (Matthews et al. 1985).

### Muscle preparation

For all experimental techniques, the extensor digitorum longus (EDL) muscle was used. This muscle is composed primarily of fast‐twitch fibers (Delp and Duan 1996; Bloemberg and Quadrilatero 2012). Mice were anesthetized with an intraperitoneal injection of sodium pentobarbital (70 mg/kg body weight) and the EDL muscles were dissected once a surgical level of anesthesia was reached. EDL muscles were immediately used for measurements of isometric contractile force, as described below. At the end of the experiments, EDL muscles were frozen rapidly in isopentane that was cooled in liquid nitrogen, and used for histochemical staining.

### Muscle contraction measurement

The force‐frequency relationship was assessed in intact EDL, as described previously (Nishi et al. 1999). Briefly, isolated EDL muscle preparations were mounted between a force transducer (UL‐100; Minebea Co., Tokyo, Japan) and fixed hock in a chamber containing Krebs solution (120 mmol/L NaCl, 5 mmol/L KCl, 2 mmol/L CaCl_2_, 1 mmol/L MgCl_2_, 1 mmol/L NaH_2_PO_4_, 25 mmol/L NaHCO_3_, and 11 mmol/L glucose) bubbled with 95% O_2_ and 5% CO_2_ at 30°C. Then isolated muscle was stimulated with 500 msec trains of current pulses at 1, 3, 10, 20, 30, 40, 50, 70, 100, 150 Hz at 1 min intervals and contractile force was measured. The preparations were stretched with a resting tension of 0.7 g and field‐stimulated with supramaximal voltage. Absolute contractile force was normalized to muscle weight.

### Muscle histology

Serial 10‐*μ*m sections were made with a cryostat (CM1510; Leica, Tokyo, Japan) at −20°C and mounted on polylysine‐coated slides. Whole sections were stained for hematoxylin and eosin, succinate dehydrogenase (SDH), fast myosin heavy chain (MHC), and Oil Red O. SDH activities in individual muscle fibers of the histological sections were examined and analyzed as described previously (Eshima et al. 2013, 2015). Mouse monoclonal antibodies that react specifically with the type IIa (1:1,000; SC‐71) and IIx (1:100; BF‐35) MHC isoforms were supplied by Developmental Studies Hybridoma Bank (University of Iowa, IA). The M.O.M. Immunodetection kit (Vector Laboratories, Burlingame, CA) and Vectastain ABC kit (Vector Laboratories) were used to reveal the immunohistochemical reaction, according to the manufacturer's instructions. Intracellular myocellular lipid (IMCL) levels were assessed by Oil Red O staining (Koopman et al. 2001; Shortreed et al. 2009). The cross‐sectional areas, SDH activities, and IMCL levels were measured by tracing fiber outlines of approximately 169 fibers per section from muscle sections. The images were digitized as gray‐level pictures. Each pixel was quantified as one of 256 gray levels and then automatically converted to an optical density using ImageJ software.

### Western blotting analysis

Western blotting was performed to determine protein expression levels in EDL muscles. The EDL muscles were excised and snap frozen in liquid nitrogen. Then, muscles were homogenized in ice‐cold RIPA lysis buffer (20 mmol/L Tris [pH 7.5], 140 mmol/L NaCl, 1 mmol/L EDTA, 50 mmol/L NaF, 1% Nonidet P‐40) containing the Halt^™^ Protease and Phosphatase Inhibitor Cocktail (50 *μ*L per mL). Homogenates were centrifuged at 20,400 *g* for 15 min at 4°C. Supernatant proteins were then quantified using the Pierce 660‐nm protein assay reagent (Thermo Fisher Scientific, Waltham, MA). Samples (seven samples were loaded onto each gel) were then electrophoresed on Mini‐PROTEAN gels (4–15% and 4–20%; Bio‐Rad Laboratories, Tokyo, Japan) and then transferred to polyvinylidene fluoride membranes, blocked with skim milk, and incubated overnight with the following primary antibodies: anti‐type 1 ryanodine receptor (RyR1) antibody 34C (Thermo Fisher Scientific, MA3‐925); anti‐dihydropyridine (DHPR) antibody 20A (Abcam, ab2864); anti‐calsequestrin (CSQ) antibody VIIID12 (Thermo Scientific, MA3‐913); anti‐SR Ca^2+^‐ATPase 1 (SERCA1) antibody IIH11 (Thermo Scientific, MA3‐911); anti‐parvalbumin (PV) (Abcam, ab32895), anti‐*α*‐tubulin antibody GT114 (GeneTex) at 4°C. Then, membranes were incubated with the appropriate secondary antibody conjugated to horseradish peroxidase and were enhanced by SuperSignal West Dura extended duration substrate (Thermo Fisher Scientific) and quantified by densitometry (C‐DiGit, LI‐COR Biosciences, Lincoln, NE). Protein levels were normalized by *α*‐tubulin expression levels and were expressed relative to the control mice fed a standard diet (control; CONT) group samples.

Extraction of myofibrils was performed as previously described (Tsika et al. 1987; Kanzaki et al. 2010). Aliquots of myofibrillar extracts proteins were subjected to electrophoresis and stained with Coomassie brilliant blue. Images of gels were acquired using ImageQuant LAS‐3000 (GE Healthcare Life Sciences, Tokyo, Japan). The content of myosin heavy chain (MHC) or actin proteins was evaluated densitometrically using ImageJ. Immunoblots for fast troponin T isoform (fast‐TnT) and slow troponin T isoform (slow‐TnT) proteins were detected as described above, using primary antibodies [anti‐fast TnT antibody H222, (Santa Cruz Biotechnology, sc‐20643) and anti‐slow TnT antibody H55, (Santa Cruz Biotechnology, sc‐28269)]. Protein levels were expressed relative to the CONT group samples.

### Statistical analysis

Values are expressed as means ± SE. Statistical analyses were performed in Prism version 5.0 (GraphPad Software, San Diego, CA). Two‐way ANOVA were used to identify group differences in IPGTT and muscle force. Bonferroni‐adjusted post hoc tests were performed to compare CONT and HFD groups at each time and frequency point. Unpaired t‐tests (two‐tailed) were used for physical characteristics, relative protein levels and histological data. A *P* < 0.05 was considered to indicate a statistically significant difference between two groups.

## Results

### Effect of a short and long‐term HFD on physical characteristics and metabolic parameters

Consumption of a HFD for 4 weeks (4‐week HFD) and 12 weeks (12‐week HFD) resulted in a significant increase in body weight and fat mass compared with control (CONT) groups (Table 1). However, the weights of the EDL were unchanged even after a 4‐week or 12‐week HFD compared with the CONT groups. Thus, the ratios of EDL weight to body weight were significantly decreased after a 4‐week and 12‐week HFD.

**Table 1:** Physical characteristics of CONT and HFD mice

	Body weight (g) Start of experiment	End of experiment	Muscle weight (mg)	Muscle weight/body weight (mg/g)	Fat mass (g)	Insulin (ng/mL)	Blood glucose (mg/dL)	HOMA‐IR (µU/mL*mg/dL/405)
4 week								
CONT	24.52 ± 0.33 (8)	27.65 ± 0.80 (8)	12.44 ± 0.52 (8)	0.45 ± 0.01 (8)	0.55 ± 0.08 (4)	0.27 ± 0.02 (3)	62.13 ± 3.00 (8)	1.21 ± 0.11 (3)
HFD	24.53 ± 0.26 (7)	35.54 ± 1.12 (7) b	12.81 ± 0.31 (7)	0.37 ± 0.01^b^ (7)	2.26 ± 0.10 (5) b	0.87 ± 0.20 (6) a	85.57 ± 3.67 (7)	4.81 ± 0.12 (6) a
12 week								
CONT	24.79 ± 0.32 (16)	32.07 ± 0.46 (16)	13.96 ± 0.36 (16)	0.44 ± 0.02 (16)	1.04 ± 0.04 (8)	0.62 ± 0.15 (8)	72.43 ± 4.04 (7)	2.75 ± 0.51 (7)
HFD	24.22 ± 0.35 (18)	48.39 ± 0.81 (18) b	13.83 ± 0.46 (18)	0.28 ± 0.01 (18) b	2.35 ± 0.16 (10) b	1.79 ± 0.42^b^ (8)	104.57 ± 4.92 (7) b	12.96 ± 3.46 (7) b

Number in parentheses indicate the total number.

a *P* < 0.05.

b *P* < 0.01.

**Figure 1:**
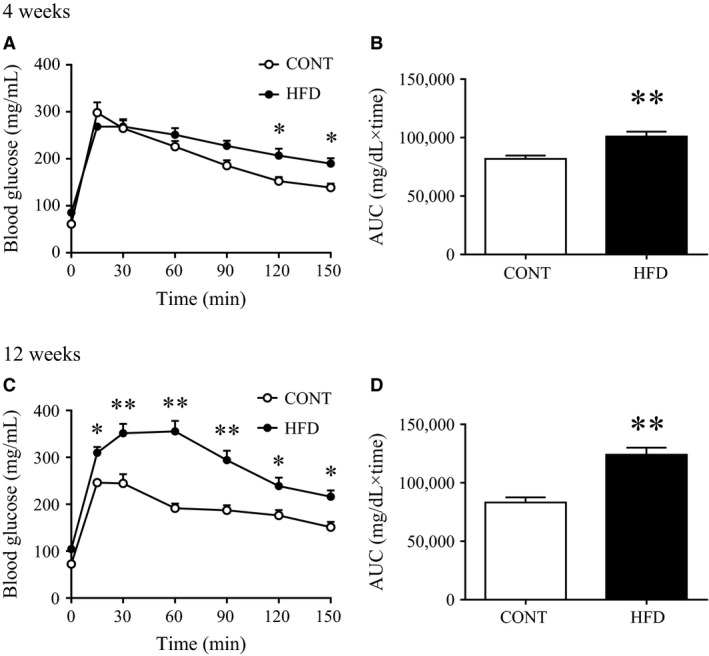
Glucose tolerance after 3 and 11 weeks on a high‐fat diet. The intraperitoneal glucose tolerance test (IPGTT) was performed after an overnight fast (16 hrs), 1 week before harvest (A: at 3 weeks; C: 11 weeks). Area under the curve (AUC) of the IPGTT test results (B: from the results in A; D: from the results in C). Values shown are means ± SE (*n *=* *7–8). *Significant difference between the control (CONT) and high‐fat diet (HFD) groups (*P <* *0.05, **P <* *0.01).

Fasting insulin levels were slightly increased after the 4‐week HFD, whereas fasting blood glucose levels were not altered (Table 1). However, both fasting glucose and insulin levels were significantly increased after a 12‐week HFD, suggesting the development of insulin resistance between 4 to 12 week on a HFD (Table 1). In fact, HOMA‐IR, a surrogate marker of insulin resistance, was consistently increased after both the 4‐week and 12‐week HFD (Fig. 1). Similarly, IPGTT performed 1 week before sacrifice showed that a 3‐week HFD slightly impaired glucose tolerance (Fig. 1, A and B) and a HFD for 12 week significantly exacerbated glucose intolerance during IPGTT (Fig. 1, C and D). These data suggested that a 12‐week HFD induces insulin resistance and impairs glucose tolerance, whereas a 4‐week HFD only moderately alters these metabolic parameters.

### Effect of short‐term and long‐term HFD on muscle contractile function

**Figure 2:**
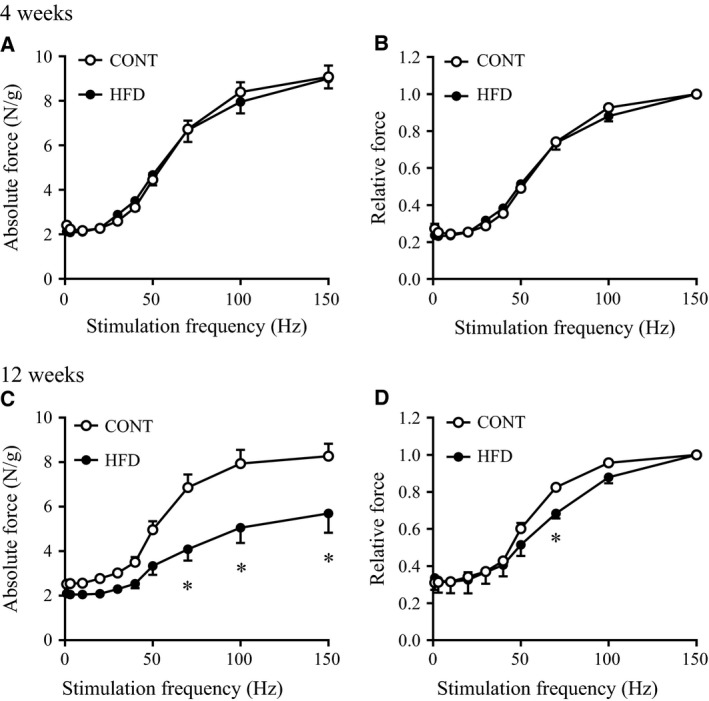
Force‐frequency relationship in fast‐twitch skeletal muscle of mice after a high‐fat diet. Isometric tension at several frequencies was determined in EDL muscles from control (CONT) and high‐fat diet (HFD)‐fed mice. Absolute force per muscle mass (A: 4 weeks; C: 12 weeks) and relative force calculated by each value normalized to the maximum force (B: 4 weeks; D: 12 weeks) were represented by the mean ± SE (*n *=* *5–7 from 5 mice). *Significant difference between CONT and HFD groups (*P *<* *0.05).

**Figure 3:**
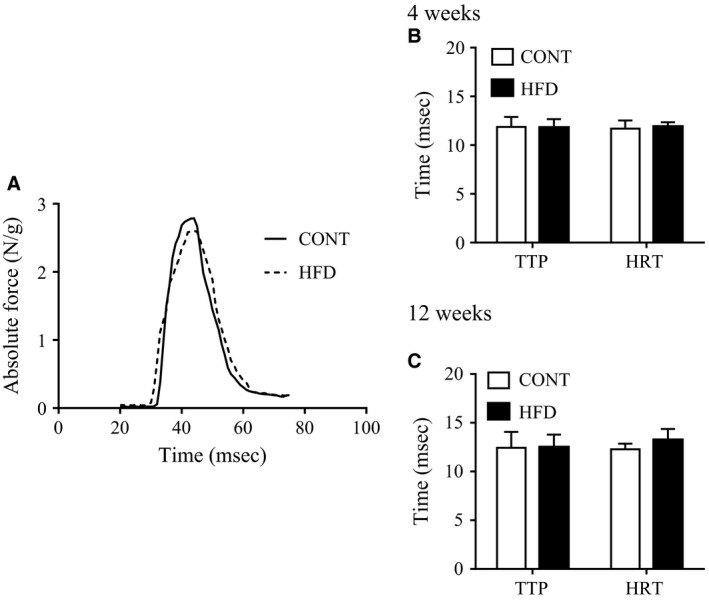
Force velocity in fast‐twitch skeletal muscle after a high‐fat diet. (A) Representative records of twitch contractile force in EDL muscles from a control mice (CONT, solid line) and a 12 week high‐fat diet‐fed mice (HFD, dashed line). Time to peak force (TTP) and half‐relaxation time (HRT) of muscle twitches in CONT and HFD mice (B: 4 weeks; C: 12 weeks). Values shown are means ± SE (*n *=* *6–10 from 5 mice).

There were no significant differences in the relative or absolute twitch forces of the EDL muscles between either of the HFD groups and the CONT group (Fig. 2). In addition, the relative and absolute tetanic force production upon electrical stimulation in EDL muscles were unchanged in the 4‐week HFD group compared with the CONT group (Fig. 2, A and B). In contrast, relative and absolute tetanic force production in EDL muscles were lower in the 12‐week HFD group than in the CONT group (Fig. 2, C and D), particularly at stimulation frequencies from 70 to 150 Hz for absolute value (Fig. 2C). The time to peak force and the half‐relaxation time in twitch stimulation did not differ between both HFD groups and the CONT group (Fig. 3). These data indicate that a long‐term (12 week) HFD, but not a short‐term (4 week) HFD, impairs muscle contractile force, whereas both twitch and relaxation speed were not altered.

### Effect of short‐term and long‐term HFD on muscle morphology

**Figure 4:**
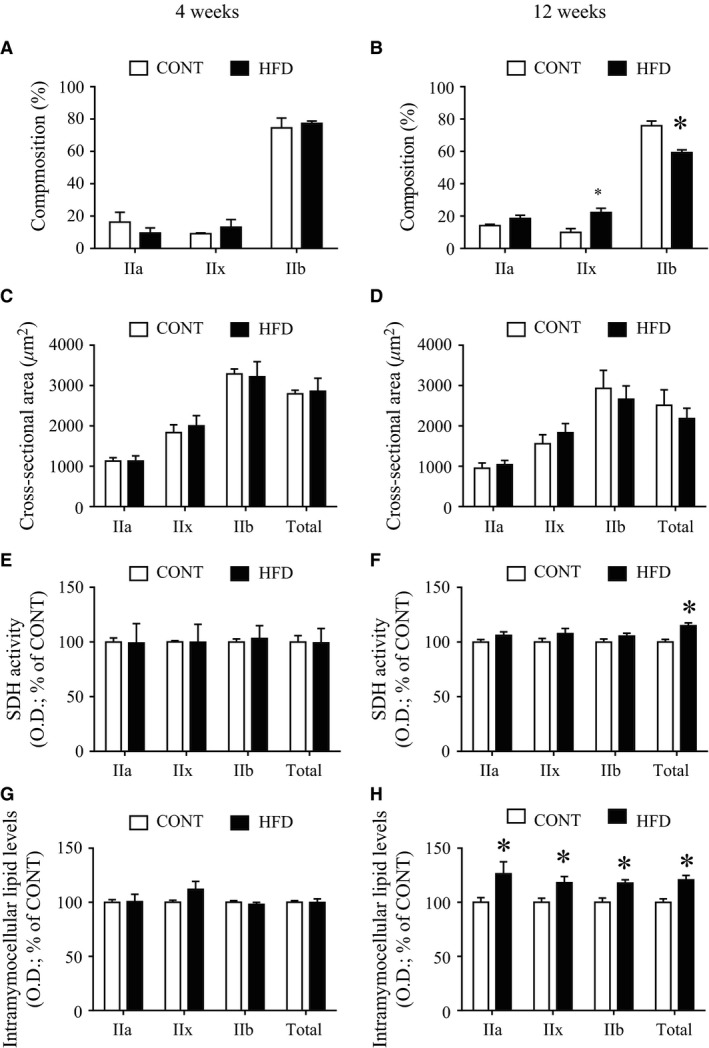
Morphometric changes of muscles after a high‐fat diet. Composition (A and B), cross‐sectional area (C and D), succinate dehydrogenase (SDH) activity (E and F), and intramyocellular lipid levels determined by Oil Red O staining (G and H) of extensor digitorum longus muscles in control mice (CONT) and high‐fat diet‐fed mice (HFD; left: 4 weeks; right: 12 weeks). succinate dehydrogenase (SDH) and Oil Red O staining are graphically represented by arbitrary units of optical density (O.D.), with higher values representing more intense staining, normalized to a percentage of the overall mean control value for each graph (% of CONT). Values are means ± SE (*n *=* *3–7). *Significant difference between CONT and HFD groups (*P *<* *0.05).

In EDL muscles from the 12‐week HFD group, but not the 4‐week HFD group, there was an increased percentage of MHC type IIa/x fibers, mainly at the expense of decreased type IIb fibers, compared with those from the CONT group (Fig. 4, A and B), whereas total cross‐sectional areas were unchanged in both HFD groups (Fig. 4, C and D). SDH activity was unchanged in the 4‐week HFD group (108.8 ± 8.8 O.D. in the 4‐week HFD vs 98.9 ± 3.4 O.D. in the CONT group; *P *>* *0.05; Fig. 4E), but the 12‐week HFD group showed evidence of significantly increased SDH activity in total fiber analysis (*P *>* *0.05; Fig. 4F). Furthermore, in the 12‐week HFD, but not in the 4‐week HFD group (*P *<* *0.05; Fig. 4G), increased IMCL levels were observed in all type II fiber types compared with the CONT group (*P *<* *0.05; Fig. 4H).

### Effect of short‐term and long‐term HFD on proteins associated with muscle contraction

**Figure 5:**
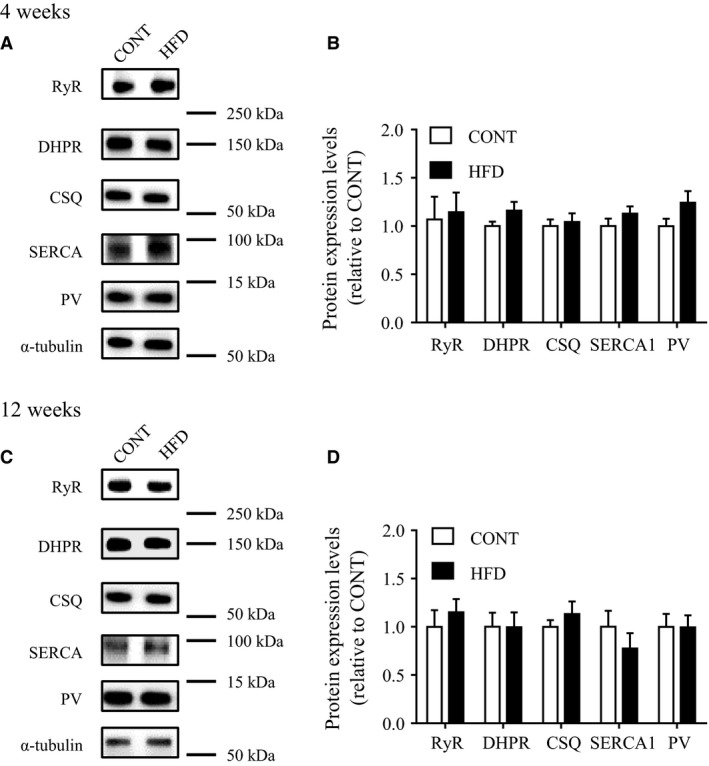
Expression levels of muscle calcium‐regulated proteins in mice after a high‐fat diet. Protein levels in control mice (CONT) and high‐fat diet‐fed mice (HFD; upper panel: 4 weeks; lower panels: 12 weeks). Left: representative western blots of RyR, DHPR, CSQ, SERCA1 and PV proteins (A: 4 weeks; C: 12 weeks). A protein expression levels expressed relative to the value of CONT mice (B: 4 weeks; D: 12 weeks). Values are means ± SE (*n *=* *7).

**Figure 6:**
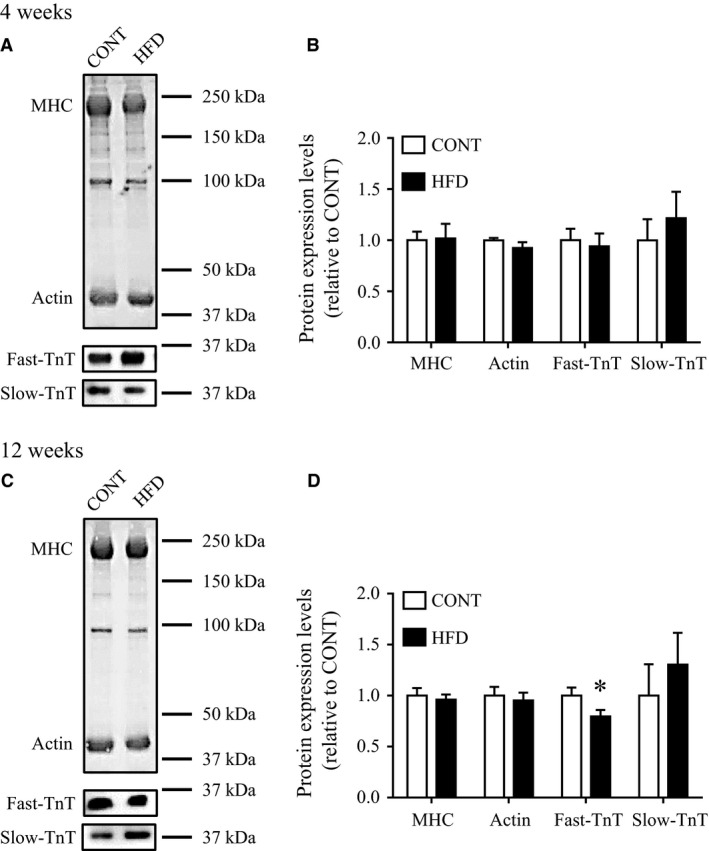
Expression levels of myofibrillar proteins in mice after a high‐fat diet. Electrophoretic separation of myofibrillar proteins by polyacrylamide gradient (4–15%) gel in control mice (CONT) and high‐fat diet (HFD)‐fed mice (A: 4 weeks; C: 12 weeks). Representative western blots of fast‐troponin T (fast‐TnT) and slow‐troponin T (slow‐TnT) were also presented. Protein content of fast‐TnT and slow‐TnT were normalized by total myofibrillar proteins expression levels and expressed relative to the value of CONT mice (B: 4 weeks; D: 12 weeks). Values are means ± SE (*n *=* *4–6). *Significant difference between CONT and HFD groups (*P *<* *0.05).

Proteins playing an important role in skeletal muscle Ca^2+^ regulation were analyzed by western blotting. No significant differences in the protein expression levels of RyR, DHPR, CSQ, SERCA1, and PV were noted between both HFD groups and the CONT group (Fig. 5). Next, we assessed the expression levels of myofibrillar proteins in HFD groups and the CONT group. Neither the MHC nor actin content were altered in HFD groups compared with CONT groups (Fig. 6). We also evaluated troponin T, a component of the regulatory troponin complex that plays a role in Ca^2+^ sensitivity and contractile function in skeletal muscle (Ogut et al. 1999). Expression levels of fast‐TnT was approximately 20% lower in the 12‐week HFD than in the CONT group (*P *<* *0.05), but not in the 4‐week HFD group. Expression levels of slow‐TnT was relatively higher after 12‐week HFD compared with CONT (Fig. 6).

## Discussion

In this study, we investigated the effects of a short‐term and long‐term HFD on morphological and functional features of fast‐twitch dominant muscle. The main findings were the following: (1) A long‐term (12 week) HFD, but not a short‐term (4 week) HFD, impaired tetanic contractile force in fast‐twitch dominant muscle independently of muscle weight loss; (2) This decreased contractile force after a long‐term HFD was associated with changes in muscle fiber‐type composition, SDH activity, insulin resistance, IMCL accumulation, and decreased fast‐TnT expression levels.

Although it has been reported that muscle strength normalized to fiber size is decreased in obese humans (Choi et al. 2016), a rodent model of obesity created by a short‐term HFD in mice did not result in impaired muscle contractile force in fast‐twitch muscles (Shortreed et al. 2009; Thomas et al. 2014). Consistently, we found that a short‐term HFD (4 week) did not significantly alter contractile force (Fig. 2, A and B); however, we demonstrated for the first time that a significant decrease in tetanic contractile force in fast‐twitch dominant muscles occurs after a long‐term HFD (12 week) compared with the control group (Fig. 2, C and D). Muscle weight generally increases with body weight gain; however, we observed similar muscle weights and cross‐sectional areas of the EDL muscle after the 4 or 12 week HFD, compared with the CONT group. The sustained muscle weight after a HFD suggests that decreased muscle contractile force after a long‐term HFD is not due to muscle volume loss, but due to impaired muscle contractile force.

Mammalian skeletal muscle strength largely depends on muscle fiber type (Gilliver et al. 2009; Schiaffino and Reggiani 2011). In particular, in mouse skeletal muscle, MHC type IIb fibers strongly contribute to muscle contractile force rather than type IIa/x and type slow fibers, classified according to MHC composition. For example, unloaded shortening velocity in type IIx, IIa, and slow fibers are significantly lower than that in type IIb fibers by 34%, 46%, and 74%, respectively (Nyitrai et al. 2006). In addition, Andruchov et al. (2004) demonstrates that the MHC type I fibers exhibited a smaller specific force than MHC type II fibers in mouse skeletal muscles. Consistent with previous studies (Shortreed et al. 2009; Thomas et al. 2014; Mastrocola et al. 2015), we observed an increased percentage of type IIx fibers as a result of a decrease in type IIb fibers in EDL muscle in long‐term HFD‐fed mice, but these changes were not observed in short‐term HFD‐fed mice (Fig. 4, A and B). These results suggest that a change in the ratio of fiber types may be one of the mechanisms resulting in decreased muscle contractile force associated with a long‐term HFD. However, causal relationship between fiber type composition and muscle contractile force remains to be determined.

Our data showed a decrease in fast‐TnT protein expression levels in long‐term HFD‐fed mice, but not in short‐term HFD‐fed mice (Fig. 6). TnT plays a role in regulating the conformational changes in thin myofilaments during excitation–contraction coupling, and contribute to contractile force in skeletal muscle (Wei and Jin 2011; Wei et al. 2014). Fast‐TnT and slow‐TnT are mainly expressed in fast‐twitch and slow‐twitch dominant muscles, respectively (Brotto et al. 2006; Wei and Jin 2016), and different patterns of TnT expression may partially explain the difference in muscle contractile force between fast and slow muscle fibers (Wang et al. 2001). It has been reported that HFD induces not only a shift from type II/glycolytic toward type II/oxidative muscle fibers, but also a shift in TnT isoform expression from fast to slow (Schilder et al. 2011; Ciapaite et al. 2015). Thus, in this study, the decrease in fast‐TnT protein levels after a long‐term HFD may partially reflect the fiber type change, and may contribute to impaired muscle contractile force.

Many studies have demonstrated that control of muscle contraction and relaxation speeds are critically dependent on effective SR Ca^2+^ handling (Allen et al. 2008). A previous study demonstrated impaired Ca^2+^ handling during tetanic muscle contraction in the *ob/ob* mouse, which is a model of obesity (Bruton et al. 2002). Furthermore, the *db/db* mouse, a model of obesity associated with type 2 diabetes, demonstrates contractile dysfunction and the reduced expression of SERCA pump levels in skeletal muscle (Bayley et al. 2016). However, our data showed no differences in the expression of calcium‐associated proteins between the HFD groups and the CONT group (Fig. 5). Consistent with this, the time to peak contraction and to half‐relaxation in the twitch stimulation experiments did not change in either HFD group, compared with in the control group (Fig. 3). These data suggest that a 12‐week HFD may not affect SR Ca^2+^ handling, and thus the speed of muscle contraction and relaxation are maintained.

The long‐term HFD altered several metabolic characteristics in skeletal muscle. For example, the 12‐week HFD, but not the 4‐ week HFD, increased muscle SDH activity. These oxidative changes after a HFD have been reported in a previous study (Thomas et al. 2014), but the change did not result in functional benefits in muscle fatigue resistance, or whole body exercise capacity (Thomas et al. 2014). Furthermore, Turner et al. also demonstrated that increased mitochondrial enzyme activities and mitochondrial respiratory chain subunit protein levels in skeletal muscle in HFD‐induce obese rats (Turner et al. 2007). Consistently, our data demonstrated that SDH activity was increased after 12‐week HFD, which is very similar to other study (Li et al. 2016). In contrast, previous studies demonstrated that insulin resistance is closely associated with IMCL accumulation (Bachmann et al. 2001; Kakehi et al. 2016) and decreased muscle strength in nondiabetic adults (Barzilay et al. 2009). In addition, strength training improved muscle strength and whole‐body insulin sensitivity in older adults with type 2 diabetes (Brooks et al. 2007). It has also been reported that greater IMCL accumulation is closely associated with impaired muscle fiber contractile force in obese older adults (Choi et al. 2016). Consistently, in the present study, progression of the decrease in muscle contractile force during the HFD was accompanied by the accumulation of IMCL and insulin resistance. However, the mechanisms by which IMCL and insulin resistance lead to decreased muscle contractile force are unknown, and thus further studies are required to clarify this research question.

In conclusion, this study demonstrated that a long‐term HFD, but not a short‐term HFD, causes decreased muscle tetanic contractile force in fast‐twitch dominant muscle fibers independently of muscle weight loss in mice. The decreased muscle contractile force induced by the long‐term HFD in mice might be due to several morphological changes in muscles, such as fiber type composition. In the future, experiments should be performed to specifically clarify the mechanisms underlying the association between HFD and muscle contractile function.

## Conflict of Interest

The authors have no disclosures.
